# Prediction of postoperative survival of triple-negative breast cancer based on nomogram model combined with expression of HIF-1α and c-myc

**DOI:** 10.1097/MD.0000000000017370

**Published:** 2019-10-04

**Authors:** Jianxiu Cui, Hongchuan Jiang

**Affiliations:** Department of Breast Surgery, Beijing Chao-Yang Hospital, Capital Medical University, Beijing, China.

**Keywords:** c-myc, HIF-1α, nomogram, prognosis, triple-negative breast cancer

## Abstract

The aims of this study were to explore the expression of hypoxia inducible factor-1α (HIF-1α) and c-myc protein in triple-negative breast cancer (TNBC) and its clinical prognostic significance, and to establish a prediction model for postoperative survival of TNBC based on nomogram.

A total of 87 patients with TNBC at the Department of Breast Surgery, Beijing Chaoyang Hospital, Capital Medical University from January 2012 to December 2015 were enrolled in this study. Immunohistochemistry was performed to detect the expression of HIF-1α and c-myc protein in breast cancer tissues. Cox regression analyses were performed to explore the correlation between HIF-1α/c-myc expression and clinical pathological parameters as well as prognosis. Receiver-operating characteristic curve was generated for cox multivariate analysis. A nomogram was generated based on the cox multivariate analysis, and a calibration curve was prepared for the nomogram to evaluate the consistency between the predicted probability of the nomogram and the actual observed probability. The stability of nomogram model was validated with an external cohort including 39 TNBC patients.

The positive expression rates of HIF-1α and c-myc protein in breast cancer tissues were 41.4% (36/87) and 55.2% (48/87), respectively. HIF-1α expression was significantly correlated with age, tumor diameter, histological grade, lymph node status, and tumor TNM stage; c-myc expression was significantly associated with tumor diameter, histological grade, lymph node status, and tumor TNM stage. Cox univariate and multivariate analyses showed that HIF-1α and c-myc protein expression, histological grade, lymph node status, and tumor TNM stage were the independent risk factors for postoperative survival in TNBC patients. The AUC of prediction model was 0.843 (0.809–0.887). The nomogram could predict the probability of 3-year disease-free survival according to each patient's condition. The calibration curve displayed good agreement of the predicted probability with the actual observed probability, indicating that the nomogram model had great value of prediction. The external validation indicated the prediction model had good stability.

HIF-1α-positive expression, c-myc positive expression, histological grade III, lymph node positive, and TNM stage III tumors suggested that TNBC patients had a poor prognosis. This prediction model can be used to predict postoperative survival of TNBC.

## 1 Introduction

Triple-negative breast cancer (TNBC) is a special clinical subtype of breast cancer, accounting for 10% to 20% of all breast cancers.^[1,2]^ Because of its estrogen receptor, progesterone and human epidermal growth factor receptor-2 (HER-2) are negatively expressed, lacking endocrine therapy and corresponding targets for molecular targeted therapy anti-HER-2 have caused great difficulties in treatment.^[3]^ TNBC has clinical features such as high histological grade, late stage, strong tumor invasiveness, and high risk of early recurrence.^[4,5]^ The prognosis is often poor, and the 5-year survival rate is <30%.^[6]^ The biomarker of TNBC remains unclear, and finding effective biomarkers to screen patients with poor prognosis has become an urgent problem to be solved.

Several previous studies^[7–9]^ have shown that hypoxia inducible factor-1α (HIF-1α) was highly expressed in breast cancer, which was associated with increased risk of breast cancer recurrence and metastasis, chemoradiation resistance, immune escape, and poor prognosis. In many studies, c-myc, as one of the early discovery of proto-oncogenes, was also highly expressed in breast cancer and other tumors^[10–12]^; however, the expression of c-myc in TNBC and its prognostic value have not been reported. Therefore, this study examined the expression of HIF-1α and c-myc in TNBC, and explored its impact on clinicopathological parameters. Based on the nomogram, a postoperative survival prediction model of TNBC was established.

## 2 Materials and methods

### 2.1 Study patients

This study contained a development cohort and a validation cohort. The development cohort included 87 patients with TNBC at the Department of Breast Surgery, Beijing Chaoyang Hospital, Capital Medical University from January 2012 to December 2015. All patients were followed up regularly. The age ranged from 31 to 78 years, with an average of (50.4 ± 16.1) years and a median age of 50 years. Fifty cases had a tumor diameter which was >2 cm. The postoperative histopathological results showed as follows: 5 cases in grade I, 48 cases in grade II, 34 cases in grade III; 43 cases of lymph node positive; 19 cases in TNM stage I, 54 cases in TNM stage II, 14 cases in TNM stage III. In 8 patients, the proportion of genes containing Ki-67 was ≤14%, whereas in 79 patients the proportion Ki-67 containing was >14%.

The validation cohort included 39 patients with TNBC at the Department of Breast Surgery, Beijing Friendship Hospital, Capital Medical University from January 2014 to January 2016. The collection methods of clinical, pathological, follow-up data were as same as the development cohort. There was no significant difference in clinical parameters between the development cohort and validation cohort (Table 1, all *P* > .05).

**Table 1:**
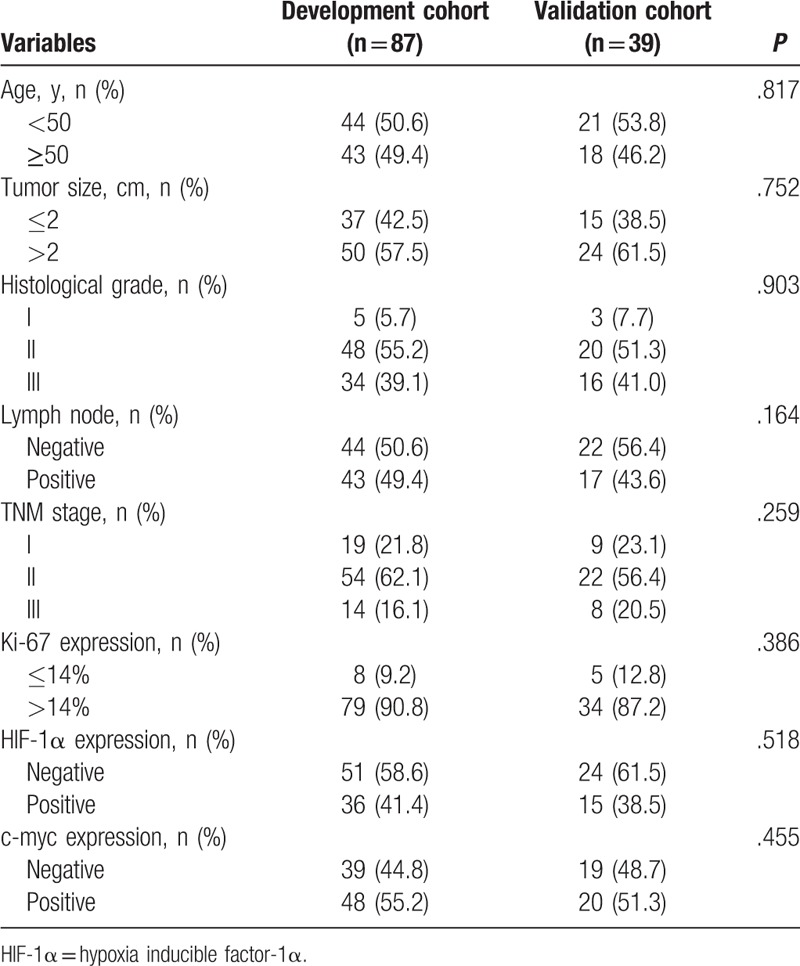
Clinical characteristics of enrolled patients in development cohort and validation cohort.

The clinical information was acquired by retrospective review of all patient medical records and approved by the Ethics Committee at Chaoyang Hospital, Capital Medical University (approval number: 2012013). Informed consent was provided by all subjects. All procedures in this study were done in accordance with the ethical standards of the institutional and/or national research committee.

### 2.2 Inclusion criteria

Patients were enrolled in this study if they met all the following criteria: patients who underwent adenocarcinoma radical surgery or breast-conserving surgery; pathological diagnosis was confirmed as TNBC; clinical data, pathological results, and follow-up data were complete; surgical pathological sections were well-preserved.

### 2.3 Exclusion criteria

Patients meeting any of the following criteria were excluded: combined with other tumors; received radiotherapy or endocrine therapy before surgery; any incomplete clinical, pathological, or follow-up data.

### 2.4 Outcome and study design

All subjects were followed up regularly and the content of the inquiry was whether there was a TNBC-related death. The outcome was with or without a TNBC-related death within 3 years. The clinical and pathological data such as age, tumor size, histological grade, lymph node status, TNM stage, and Ki-67 ratio of all patients were collected. Immunohistochemistry (IHC) staining was performed on the surgical pathological sections of patients to detect the expression of HIF-1α and c-myc.

### 2.5 Immunohistochemical staining and scoring

A proportion of surgical tissue specimens were fixed with formalin paraffin-embedded. IHC staining was performed on 4-μm thick section. Briefly, each slide was incubated with primary antibody against HIF-1α (1:100, Abcam, UK, Cat. #ab51608) or c-myc (1:200, Abcam, UK, Cat. #ab32072) overnight after a series of procedures (de-paraffin, antigen retrieval, rinse). It was followed by an incubation with the anti-rabbit IgG-HRP antibody (1:1000, Sungene Biotech, China, Cat. #LK2001) for 30 minu. The membrane was then washed five times with TBST and enriched with the brown color of DAB Enhancer (Dako). The expression of HIF-1α or c-myc was evaluated by three experienced pathologists. The “positive” result in IHC images showed that the nucleus and cytoplasm of the cells were yellow or brownish yellowes fine particles. The “negative” result showed no evidence of yellow or brownish yellow particles in nucleus and cytoplasm.

### 2.6 Statistical analysis

*χ*^2^ test and Mann–Whitney test were performed to analyze the relationship between the expression of HIF-1α, c-myc protein, and clinicopathological parameters of patients with TNBC. Cox univariate and multivariate survival analyses were used to estimate the independent factors of survival prognosis. Receiver-operating characteristic (ROC) curve and nomogram were generated based on cox regression analyses. The calibration curve was founded to evaluate the agreement of the nomogram-predicted probability with the actual observed probability. The stability of prediction model was confirmed with the validation cohort. SPSS 16.0 (SPSS Inc, Chicago, IL) was used to perform all statistical analyses and ROC curve generating. Nomogram and calibration curve were generated with R version 3.5.0 and *P* value <.05 was considered significant.

## 3 Results

### 3.1 Expression of HIF-1α and c-myc in breast tumor tissues of patients with TNBC

**Figure 1:**
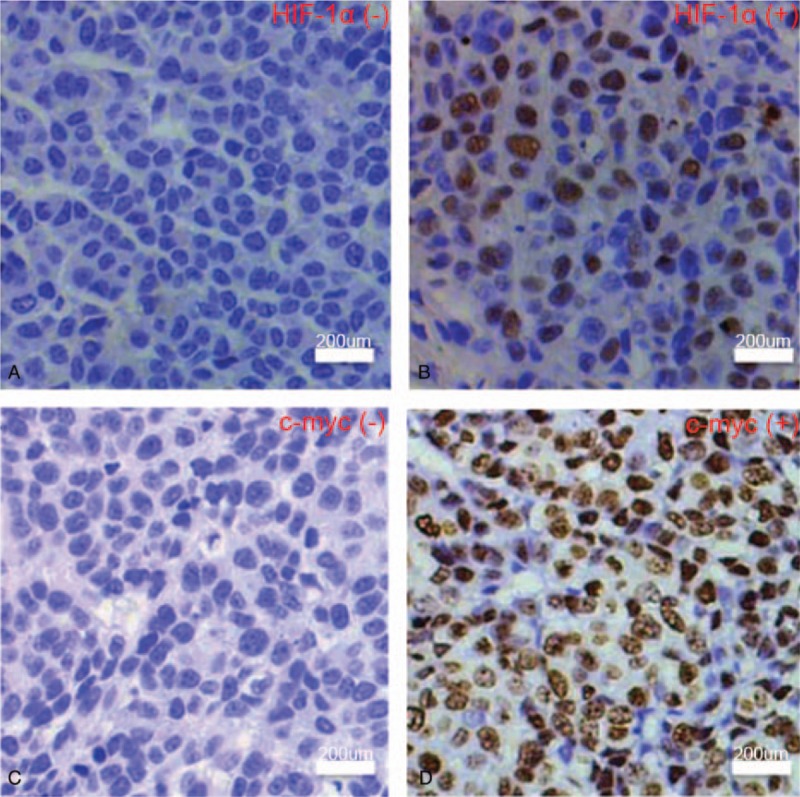
The histological morphology of HIF-1α and c-myc in triple-negative breast cancer. (A) HIF-1α negative; (B) HIF-1α positive, colored in the nucleus and cytoplasm; (C) c-myc negative; (D) c-myc positive, stained in the nucleus and cytoplasm (bar = 200 μm). HIF-1α = hypoxia inducible factor-1α.

Both HIF-1α and c-myc protein were expressed in the cytoplasm and nucleus. The positive result in IHC image showed that the nucleus and cytoplasm of the cells were yellow or brownish yellow fine particles. The negative result showed no evidence of yellow or brownish yellow particles in nucleus and cytoplasm (Fig. 1). In 87 patients, the positive expression rates of HIF-1α and c-myc protein were 41.4% (36/87) and 55.2% (48/87), respectively.

### 3.2 Association between HIF-1α, c-myc and clinicopathological features of TNBC

In the Table 2, *χ*^2^ test and Mann–Whitney test showed that HIF-1α expression was significantly correlated with age, tumor size, histological grade, lymph node status, and TNM stage (*P* < .05). The expression of c-myc was significantly correlated with tumor size, histological grade, lymph node status, and TNM stage (*P* < .05). HIF-1α expression was higher in elderly patients. HIF-1α and c-myc were significantly elevated in patients with tumor size > 2 cm, histological grade III, lymph node-positive, and TNM stage III. It meant that highly expressed HIF-1α and c-myc were closely and positively correlated with TNBC which in a high degree of malignancy. However, there was no significant relationship between HIF-1α, c-myc, and the expression of Ki-67 (*P* > .05).

**Table 2:**
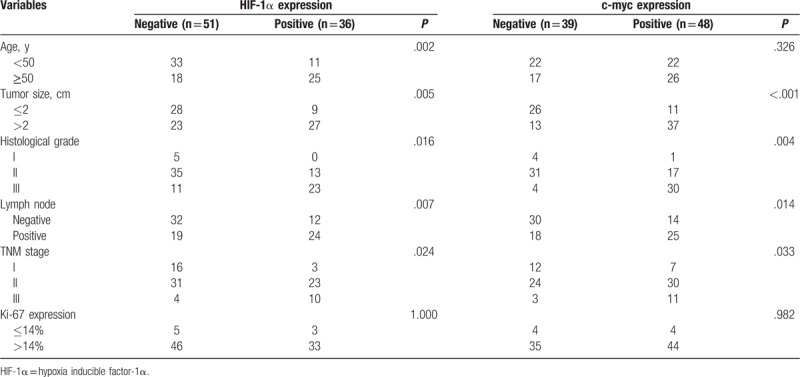
The association between HIF-1α, c-myc, and clinicopathological features of TNBC.

### 3.3 Cox univariate analysis of postoperative survival in TNBC patients

The clinical pathological variables such as age, tumor size, histological grade, lymph node status, TNM stage, HIF-1α expression, c-myc expression, and Ki-67 expression were included in Cox univariate analysis. The results (Table 3) showed age and Ki-67 were not factors influencing postoperative survival in TNBC patients (*P* > .05). Tumor size >2 cm (*P* = .033), histological grade III (*P* = .012), lymph node-positive (*P* = .024), TNM stage III (*P* = .005), HIF-1α-positive expression (*P* < .001), and c-myc-positive expression (*P* = .003) were the risk factors of postoperative survival in TNBC patients. The hazard ratios (HRs) were as follows: lymph node positive (HR = 3.011), histological grade (HR = 2.340), HIF-1α positive expression (HR = 2.027), TNM stage III (HR = 1.632), c-myc-positive expression (HR = 1.514), tumor size > 2 cm (HR = 1.465).

**Table 3:**
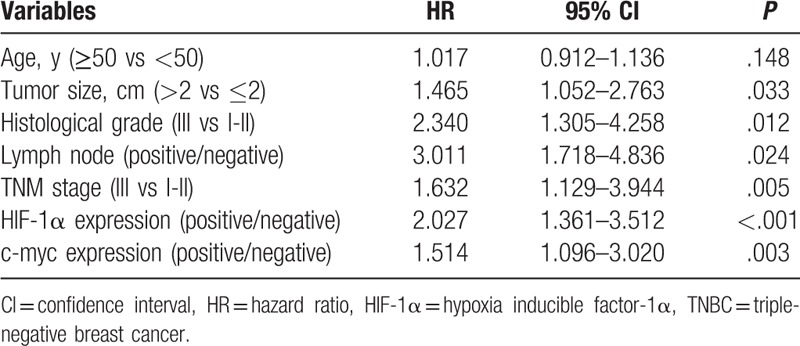
Cox univariate regression analysis of postoperative survival in TNBC.

### 3.4 Cox multivariate analysis of postoperative survival in TNBC patients

Cox univariate analysis showed tumor size, histological grade, lymph node status, TNM stage, HIF-1α expression, and c-myc expression were the factors influencing postoperative survival in TNBC patients. The results of collinearity test showed that there was no collinearity between each factor. Therefore, all the above factors were analyzed by Cox multivariate analysis. The results (Table 4) showed that histological grade III (*P* = .042), lymph node-positive (*P* = .015), TNM stage III (*P* = .028), HIF-1α-positive expression (*P* < 0.001), and c-myc-positive expression (*P* = .007) were the independent risk factors for postoperative survival in TNBC patients and could be used to predict 3-year prognosis. The HRs were as follows: lymph node status (HR = 2.893), HIF-1α-positive expression (HR = 2.215), histological grade (HR = 1.794), c-myc-positive expression (HR = 1.688), TNM stage III (HR = 1.626).

**Table 4:**
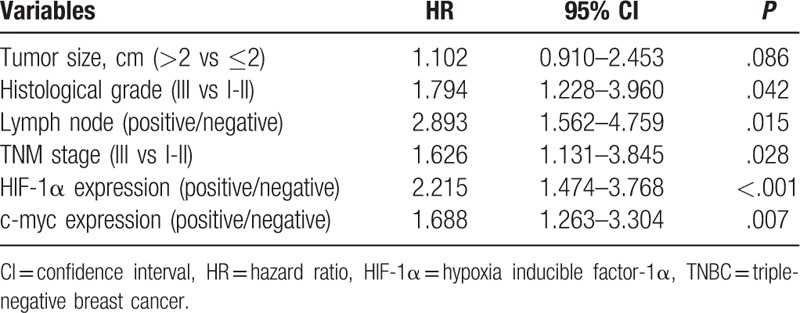
Cox multivariate regression analysis of postoperative survival in TNBC.

### 3.5 ROC curve of postoperative survival prediction model for TNBC patients

**Figure 2:**
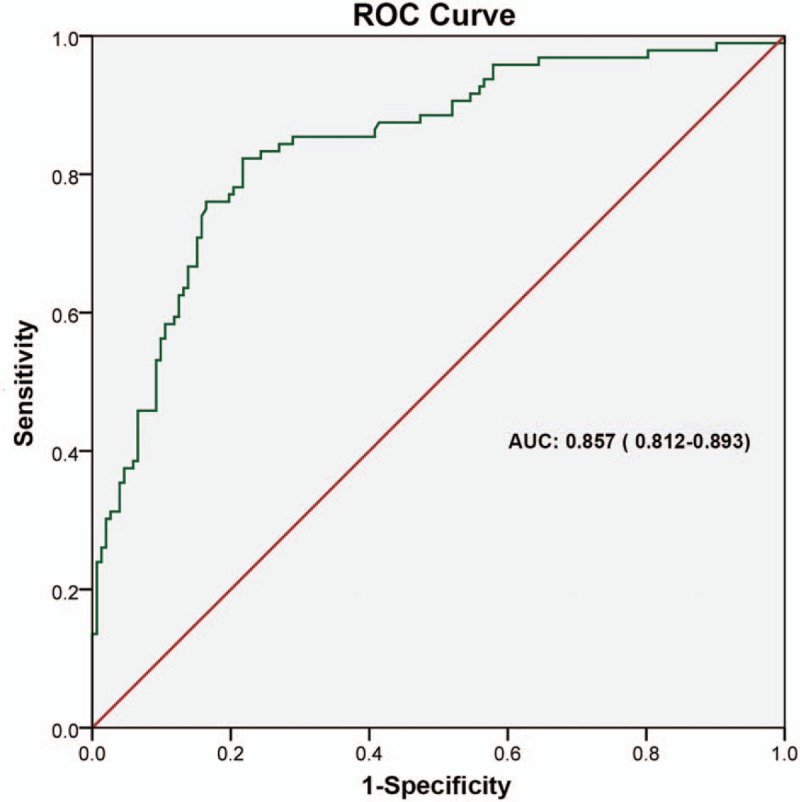
The ROC curve of postoperative survival prediction model for triple-negative breast cancer patients. The AUC was 0.857 (0.812–0.893). AUC = area under the curve, ROC = receiver-operating characteristics.

The ROC curve was performed on the outcome variable “death” using the probability of individual death which calculated based on Cox multivariate analysis. It could assess the predictive value of the model. The area under curve (AUC) was 0.857 (0.812–0.893), indicating that the model had a good predictive value (Fig. 2).

### 3.6 Nomogram of postoperative survival prediction model for TNBC patients

**Figure 3:**
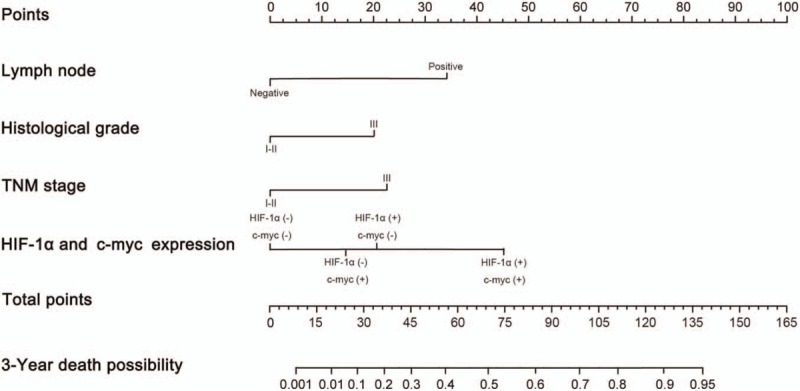
The nomogram of postoperative survival prediction model for triple-negative breast cancer patients according to histological grade, lymph node status, TNM stage, HIF-1α expression, and c-myc expression. To estimate the risk of postoperative survival, the points for each variable were calculated by drawing a straight line from a patient's variable value to the axis labelled “Points.” The score sum is converted to a probability in the lowest axis. HIF-1α = hypoxia inducible factor-1α.

Based on the Cox multivariate analysis, the 5 independent risk factors such as histological grade, lymph node status, TNM stage, HIF-1α expression, and c-myc expression could be included to generate a nomogram. In this nomogram, HIF-1α and c-myc were divided into 4 cases: both HIF-1α and c-myc were negative, HIF-1α negative and c-myc positive, HIF-1α positive and c-myc negative, both HIF-1α and c-myc were positive. According to the information of each patient (histological grade, lymph node status, TNM stage, HIF-1α expression, and c-myc expression), postoperative survival probability could be calculated directly. The clinical information of each patient was included in the nomogram for matching analysis. The sensitivity was 86.2% and the specificity was 79.4%. It also proved that the nomogram had good predictive value (Fig. 3).

### 3.7 Evaluation of postoperative survival prediction model for TNBC patients

**Figure 4:**
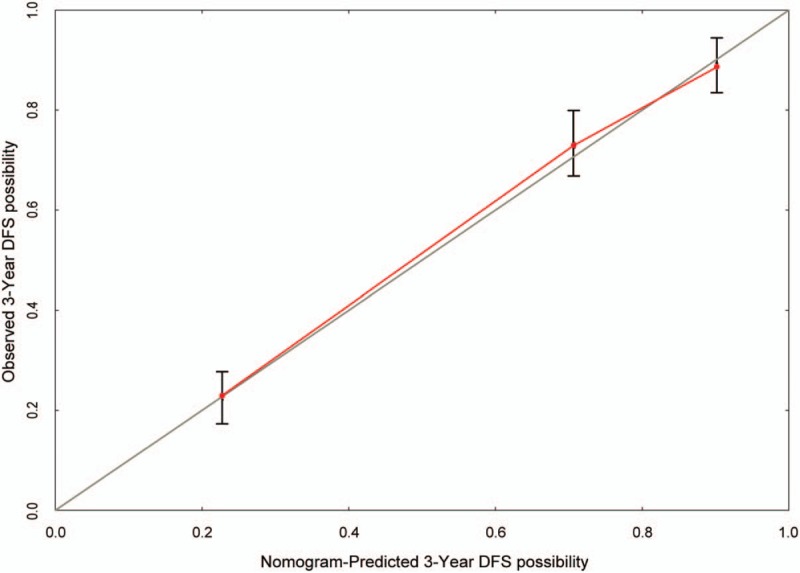
Calibration curve of postoperative survival prediction model for triple-negative breast cancer patients. The nomogram-predicted probability is plotted on the *x*-axis, and the actual probability is plotted on the *y*-axis.

A calibration curve was prepared for the nomogram to evaluate the consistency between the predicted probability of the nomogram and the actual observed probability. The calibration curve showed the predicted probability curve and the actual observation curve. The calibration curve displayed good agreement of the predicted probability with the actual observed probability, indicating that the nomogram model had great value of prediction (Fig. 4). To confirm the stability of the prediction model, external data validation was performed used another cohort. For 3-year death prediction, the sensitivity was 88.2% and the specificity was 72.7% (Table 5).

**Table 5:**
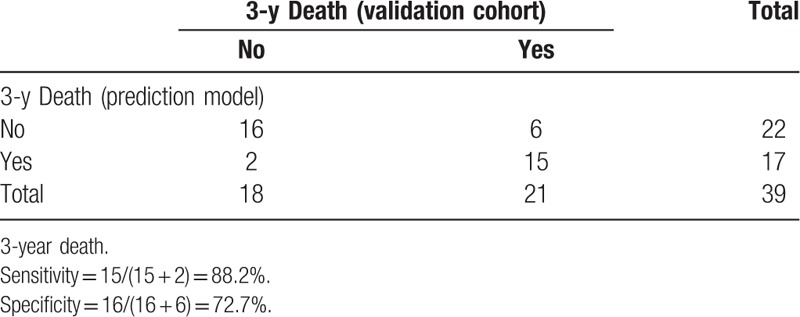
External data validation of the prediction model for postoperative survival.

## 4 Discussion

The characteristics of TNBC were poorly differentiated, invasive, and easily metastatic. The prognosis was extremely poor. Moreover, there was no specific molecular-targeted therapy.^[13,14]^ It urgently needed to find effective biomarkers.

Some literatures have reported that HIF-1α was highly expressed in many tumors including breast cancer, which may be involved in the process of apoptosis, proliferation, migration, invasion, and neovascularization of tumor cells.^[15,16]^ As a transcription factor of tumor cells against hypoxic environmental stress, HIF-1α could regulate a variety of downstream genes, thereby increasing the invasive ability of tumors and increasing the resistance of tumors to external treatment.^[17,18]^ It may be the reason that TNBC was not sensitive to radiotherapy and chemotherapy. Chen et al found that the high expression of HIF-1α and PD-L1 in TNBC suggested poor prognosis, suggesting that HIF-1α may be a risk factor for survival of TNBC.^[19]^

C-myc, one of the early discovered proto-oncogenes, was a sequence-specific transcription factor.^[20]^ Similar to HIF-1α, the *c-myc* gene was highly expressed in tumors such as breast cancer, prostate cancer, cervical, cancer and colon cancer, and the c-myc gene rearrangement occurred.^[21,22]^ C-myc was associated with tumor cell growth, apoptosis, cell cycle, tumorigenesis, and progression.^[23,24]^ Rao et al^[25]^ found that in benign hyperplasia, atypical hyperplasia, breast cancer, the gene amplification of c-myc was gradually increasing, and they believed that c-myc had been activated in the early stage of tumorigenesis and participates in the whole tumor development process. Li et al reported the relationship between c-myc and recurrence and metastasis of TNBC; they found that c-myc was highly expressed in high histological-grade TNBC, suggesting that c-myc was associated with progression of TNBC.^[26]^ But it was still unclear that the prognosis impact of c-myc on TNBC.

In this study, IHC showed the positive expression rates of HIF-1α and c-myc protein in breast cancer tissues were 41.4% and 55.2%, respectively. *χ*^2^ test and Mann–Whitney test showed that HIF-1α expression was significantly correlated with age, tumor diameter, histological grade, lymph node status, and TNM stage (*P* < .05); c-myc expression and tumor diameter, Histological grade, lymph node status, tumor TNM stage were significantly correlated (*P* < .05). HIF-1α expression was higher in elderly TNBC patients. HIF-1α and c-myc were significantly elevated in patients with tumor size > 2 cm, histological grade III, lymph node positive, and TNM stage III. It meant that high expression of HIF-1α and c-myc was closely related to high malignant TNBC. Cox univariate and multivariate analyses showed that HIF-1α-positive expression and c-myc-positive expression, histological grade III, lymph node positive, and TNM stage III were independent risk factors for postoperative survival in TNBC patients, which could be used to predict 3 years’ prognosis. Specifically, the risk of death in HIF-1α-positive expression TNBC patients was 2.215 times of that HIF-1α-negative expression TNBC patients, and the risk of death in c-myc-positive expression TNBC patients was 1.688 times of that c-myc-negative expression TNBC patients. Histological grade III and TNM staging phase III increased the risk of death by 0.794 times and 0.626 times respectively compared with histology grade 1-II and TNM stage I-II. The ROC curve was performed on the outcome variable “death” using the probability of individual death which calculated based on cox multivariate analysis and the AUC was 0.857 (0.812–0.893), indicating that the model had a good predictive value.

The nomogram could calculate the approximate probability of 3-year postoperative survival based on the clinical data of each TNBC patient. The sensitivity was 86.2% and the specificity was 79.4%, which also proved that the nomogram had higher predictive value. The calibration curve as well as external validation indicated the prediction model had good accuracy and stability.

For clinicians, once a TNBC patient with the above independent risk factors is admitted, it is necessary to evaluate the prognosis of the patient after surgery and to develop a scientific and reasonable intervention plan for the patient as soon as possible.

Of course, considering the effect of racial/ethnic differences, regional disparity in incidence of TNBC and the limitation of small amount of data, multiple-center data, more cases are needed for further study.

## 5 Conclusion

This study showed that HIF-1α-positive expression and c-myc-positive expression, histological grade III, lymph node positive, and TNM stage III tumors suggested that patients with TNBC had a poor prognosis. This predictive model can be used to predict postoperative survival of TNBC.

## Author contributions

**Conceptualization:** Hongchuan Jiang.

**Data curation:** Jianxiu Cui.

**Formal analysis:** Jianxiu Cui.

**Funding acquisition:** Hongchuan Jiang.

**Investigation:** Hongchuan Jiang.

**Resources:** Hongchuan Jiang.

**Software:** Hongchuan Jiang.

**Validation:** Hongchuan Jiang.

**Writing – original draft:** Jianxiu Cui.

**Writing – review & editing:** Jianxiu Cui.
